# An overview of walnuts application as a plant-based

**DOI:** 10.3389/fendo.2022.1083707

**Published:** 2022-12-15

**Authors:** Xingjian Zhou, Xingyu Peng, Huan Pei, Yuhan Chen, Hui Meng, Jiali Yuan, Haijing Xing, Yueying Wu

**Affiliations:** ^1^ Yunnan Provincial Key Laboratory of Molecular Biology for Sinomedicine, Yunnan University of Chinese Medicine, Kunming, Yunnan, China; ^2^ College of Basic Medicine, Yunnan University of Chinese Medicine, Kunming, Yunnan, China

**Keywords:** walnut plant base, walnut protein, walnut peptide, bioactivity, gut microbiota

## Abstract

The plant-based refers to plant-based raw materials or products that are available as the source of protein and fat. Utilization and development of walnuts as a plant-based, resulting in a high-quality protein-rich walnut plant-based product: walnut protein powder and walnut peptides. Progress in research on the application of walnuts as a plant-based has been advanced, solving the problem of wasted resources and environmental pollution caused by the fact that walnut residue, a product of walnuts after oil extraction, is often thrown away as waste, or becomes animal feed or compost. This paper reviews and summarizes the research and reports on walnut plant-based at home and abroad, focusing on the application of walnut plant-based in the preparation process (enzymatic and fermentation methods) and the biological activity of the walnut protein and walnut peptide, to provide a theoretical basis for the further processing of walnuts as a walnut plant-based. It can make full use of walnut resources and play its nutritional and health care value, develop and build a series of walnut plant-based products, improve the competitiveness of walnut peptide products, turn them into treasure, and provide more powerful guidance for the development of food and medicine health industry in Yunnan.

## Graphical Abstract

The walnuts as a Plant-Based.

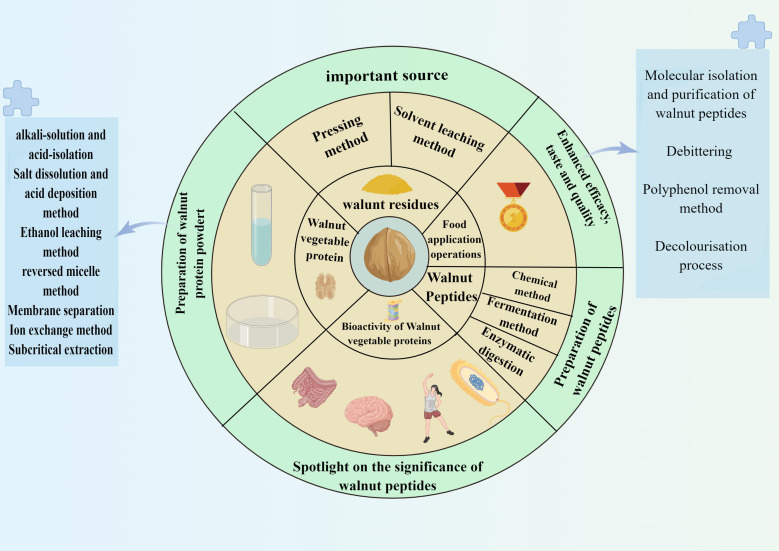

## 1 Introduction

Walnut (*Juglans regia L.*), as the medicinal and food biological resource in China, belongs to the genus Walnut in the Walnut family, rich in oleic acid, linoleic acid, α-linolenic acid, and other unsaturated fatty acids, vitamins, and proteins. It has a high nutritional value (1, 2), is known as the “longevity fruit” and the “educational fruit,” and is an important economic forest tree in China. Yunnan province is the largest walnut-producing area in China (accounting for 27.17% of the national walnut production), and Fengqing County is the main walnut-producing area in Yunnan province (3). By 2020, Fengqing County’s walnut cultivation area reaches 1,718,000 mu (mu is a municipal unit of land area in China, about 666.667 square meters), with an annual output of about 103,500 tons and an annual output value of up to 2 billion yuan, ranking first in the country for many years in terms of cultivation area and annual output (4). Walnuts are commonly used to make walnut oil because they contain 65% to 70% oil (5). However, the large number of by-products produced after their oil extraction, walnut residues, are often abandoned and even pollute the environment (6). Recently, plenty of researches have shown that walnut residues can still improve learning and memory as well as antioxidant function (7, 8), therefore, the secondary development and utilization of walnut residues need to be addressed urgently.

In recent years, there has been a boom in “plant-based products” at home and abroad. The term “plant-based products” is derived from the American Plant-Based Foods Association’s concept of “finished food products consisting of ingredients obtained from plants such as vegetables, fruits, grains, nuts, seeds and/or legumes (9)”T/CIFST 002-2021, proposed by China’s Chinese Society of Food Science and Technology in 2021, means “foods made from plant materials (including algae and fungi) or their products as a source of protein and fat, with or without the addition of other ingredients, and made by a certain process with similar texture, flavor, morphology, and other quality characteristics to those of certain foods of animal origin.”.Thus, this paper is intended to review the “walnut plant-based+” series of products using walnuts as the main raw material for plant-based food products, to provide a basis and support for increasing the added value of walnut residues and contributing to Yunnan’s biomedical industry.

## 2 Walnut residue is an important source of walnut plant-based

According to the definition of “plant-based food” by the American Plant-Based Food Association and the Chinese Society of Food Science and Technology, we consider that “walnut plant-based” can be understood as: the raw material of the walnut plant or its products that serve as a source of protein and fat, including walnut kernels and their Walnuts and their by-products after oil extraction (walnut residue). One of them, walnut kernels, is commonly used in the preparation of walnut oil and nuts and is widely used, while walnut residues are often abandoned as a by-product of walnut oil extraction, and even pollute the environment. Hence, the secondary use and exploitation of walnut residues as the main raw material for “walnut plant-based+” products is promising.

### 2.1 Walnut residue is one of the most potential “walnut plant-based+”

**Figure 1:**
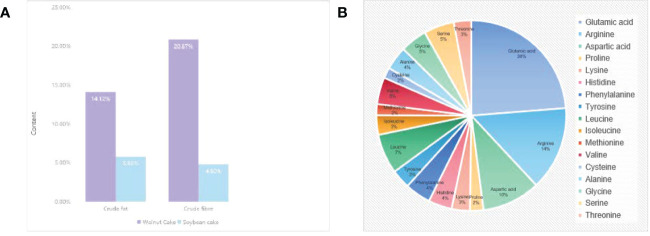
The main component of walnut residue. **(A)** Comparison of crude fat and crude fiber in walnut residue and soya residue. **(B)** Composition and content of amino acids in walnut residue.

It’s confirmed that walnut residues contain a variety of nutrients such as protein, fat, inorganic salts, vitamins, and fiber (10). The crude fat, crude fiber, and crude fiber in walnut residues are higher than those of similar nut residues, and of these, 143.4% and 334.8% are higher respectively compared to soybean residue (**Figure 1A**). The vitamin D3 content is up to 139,000 IU/kg, much higher than most nut residue (11). Besides, walnut residues have 18 amino acids, glutamic acid (21.30%~21.70%), arginine (13.60%~15.20%), and aspartic acid (10.20%~10.50%) are the main amino acids (12) (**Figure 1B**), which shows that walnut residues have high nutritional value and is a more promising “walnut plant-based +”. It can be applied in the development of walnut plant-based foods.

### 2.2 Walnut residue is often obtained by the means of pressing and solvent leaching

**Figure 2:**
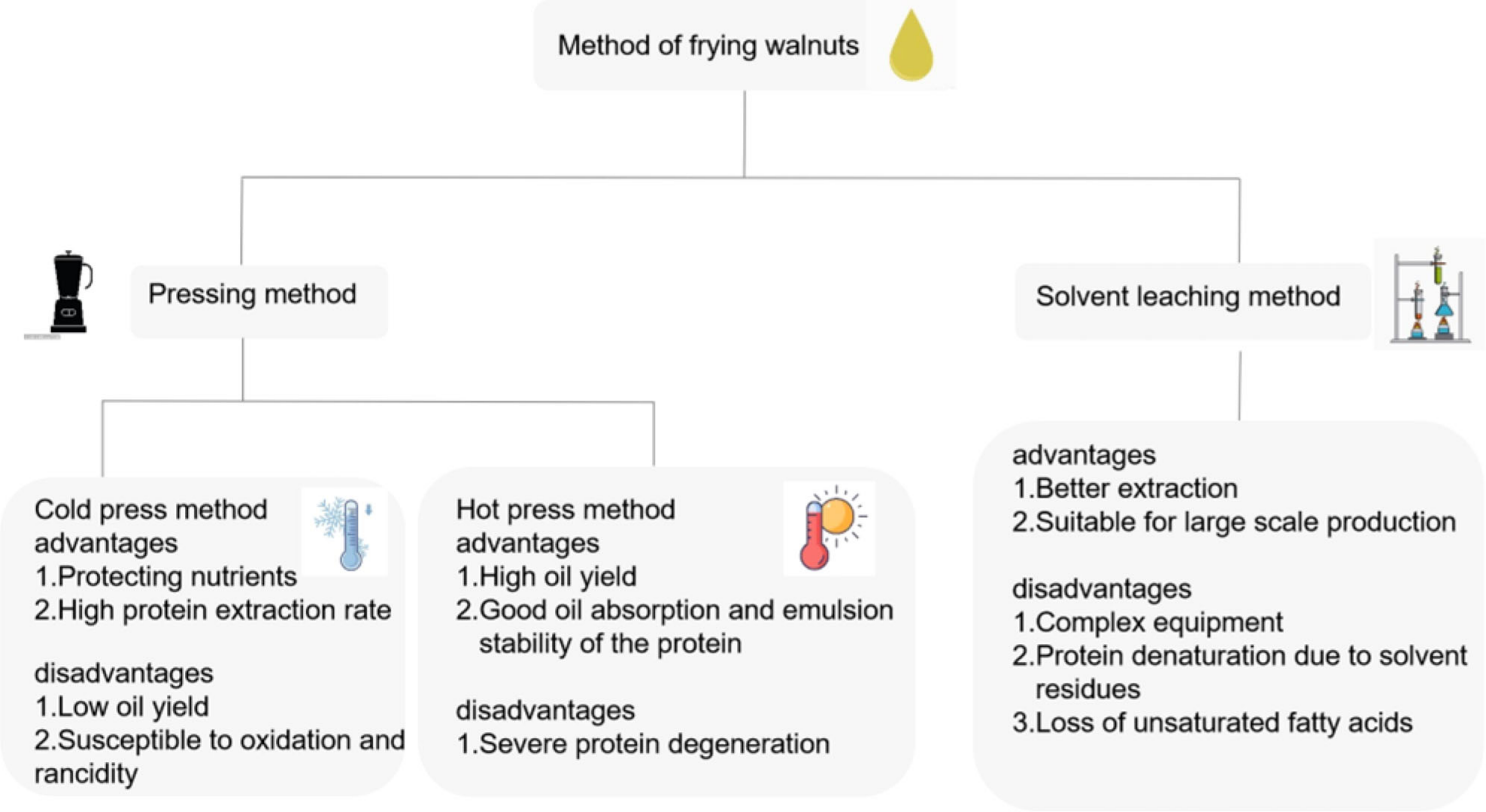
Advantages and disadvantages of the press and solvent leaching methods.

At present, the major methods of walnut oil extraction in China include pressing and solvent leaching (13). The pressing method (14) uses mechanical pressing to squeeze the oil out of the walnut kernels. The pressing methods are divided into cold pressing and hot pressing, the cold pressing method is carried out at a low temperature, without the use of chemical materials to refine the oil and meet the edible standard (13, 15). It is currently the most used method in China (16), and the obtained walnut residues can better maintain the physical properties and nutrients of walnut residues, and also has a higher protein extraction rate (6), and the protein extracted from walnut cake dross has better solubility, emulsification, and water absorption, and yet requires higher cost and lower oil output than the hot pressing method, and is prone to oxidative rancidity. The oil output of the hot pressing method is higher, and the oil absorption and emulsion stability of the protein extracted from the walnut residues are better, while the oil extraction temperature is higher, and 30% of the walnut shells are also present during the extraction process, making the composition of the obtained walnut residues complex in composition and serious protein denaturation. The organic solvent leaching method (17) is a way to extract the oil in walnut kernels with organic solvents using the extraction principle. The extraction effect is better and suitable for large-scale production, but the equipment is complicated and the solvent residue leads to protein denaturation and the loss of unsaturated fatty acids. In summary, pressing is the most commonly used method for extracting walnut oil. The properties of the walnut residues obtained from different extraction methods vary, and the researcher selects the extraction method according to the purpose of the study and the actual needs of the product development (**Figure 2**).

## 3 Walnut plant-based food

### 3.1 Walnut protein powder

**Figure 3:**
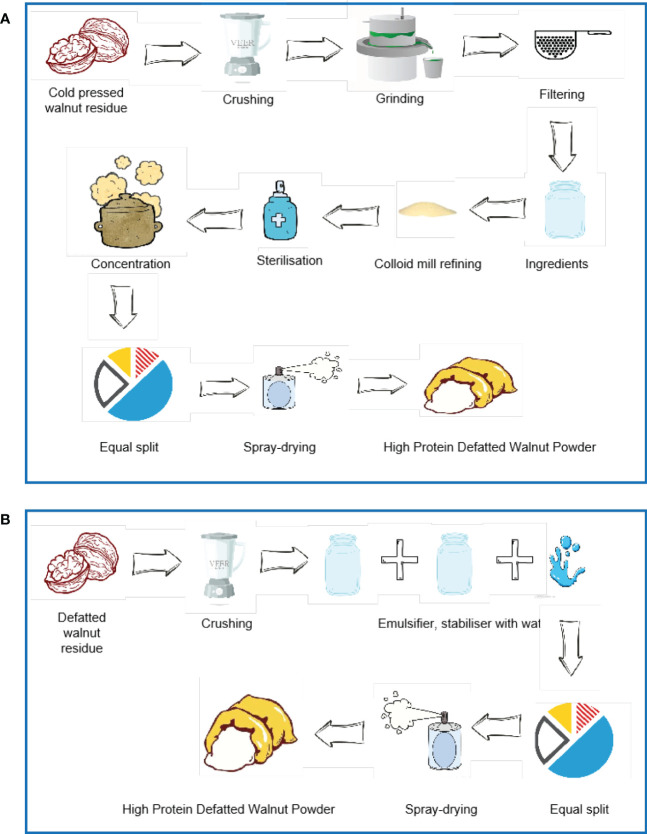
There are two main ways of preparing low-fat walnut protein powder. **(A)** The method of using the cold-pressed walnut residue as raw material. **(B)** The method of using Xinjiang high-quality walnut defatted residue as raw material.

As a plant-derived protein powder, walnut protein powder is now widely used in the food sector. Walnut protein powder is mainly a high-protein product obtained by drying and micronizing the by-products produced after the extraction of oils and fats. Walnut powder is mainly divided into two types, which include full-fat and low-fat. The low-fat walnut powder contains less oil and therefore has a longer shelf life, while the full-fat walnut powder has a high oil content and therefore has a shorter shelf life. Therefore, low-fat walnut powder is popular in the market (18). There are two main ways of preparing low-fat walnut protein powder, such as using the cold-pressed walnut residue and Xinjiang high-quality walnut defatted residue as raw material (**Figure 3**) (19). Walnut phycobilisome is weakly soluble (5) and its emulsification is positively correlated with solubility and is also influenced by the concentration of walnut phycobilisome, temperature, pH, and salt ion levels (10). Yi’s study showed (20) that the solubility, emulsification, and emulsion stability of Walnut vegetable proteins were lower at around pH 5.0. Gao’s study (21) confirmed that Walnut vegetable proteins have low emulsification near the isoelectric point and rise in emulsification away from the isoelectric point. Based on the different properties, Walnut protein powders are divided into different categories (**Table 1**).

**Table 1:** Classification of Walnut protein powders.

**Basis of classification**	**Category**			**Reference**
Protein content	Walnut protein powder (protein content < 60%)	Walnut protein concentrate (protein content > 70%)	Walnut isolate (protein content > 90%)	(10)
Oil and fat content	Full fat powder	Semi-skimmed powder	Degreasing powder	(22)
Solubility	Gluten (more than 70%), clear protein, globulin, alcoholic soluble protein			(23)

#### 3.1.1 Walnut protein is often prepared by alkali solution and acid-isolation

**Figure 4:**
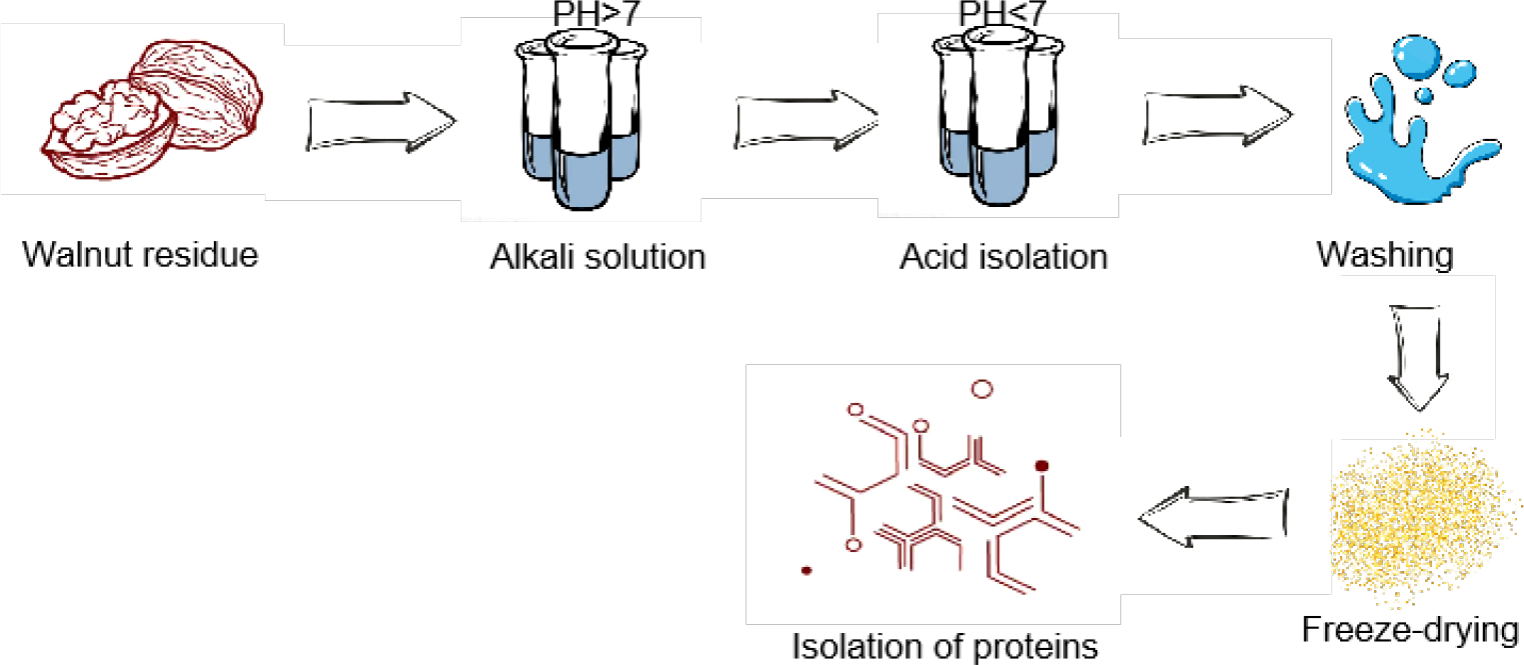
Process flow diagram for the preparation of Walnut vegetable protein.

Walnut protein powder is mainly prepared using walnut residues. Commonly used methods for the preparation of Walnut protein powder include salt-soluble acid precipitation, dilute acid precipitation, alkali-soluble acid precipitation, enzymatic digestion, reverse micellar method, membrane separation, ion exchange, and physically assisted methods (24) (**Table 2**). Among all these methods, the alkali-solution and acid-isolation method has the advantages of high purity of the isolated protein and good quality of the product and is the main and commonly used preparation method, which has been widely used in the preparation and practical production of walnut isolated protein at domestic and overseas (**Figure 4**). However, the effect of acid and alkali made the prepared Walnut protein poor in organoleptic properties, showing a brownish color and requiring further decolorization (5, 24). Yang (28) investigated the effects of four factors on the protein extraction rate, NaOH solution concentration, material-to-liquid ratio, extraction temperature, and extraction time using the alkali-solution and acid-isolation methods. The optimum extraction conditions for alkali-soluble pecan kernel protein were determined as “NaOH concentration 0.02 mol/L, material-to-liquid ratio 1:30 (g/mL), extraction temperature 50°C, extraction time 1.5 h, and an isoelectric point 4.5”.To improve the yield of Walnut vegetable proteins, auxiliary extraction techniques such as ultrasonic technology and subcritical water can be used, with mild reaction conditions and easy operation (27, 29, 30). As can be seen, each of the methods for preparing Walnut protein powder has its advantages and disadvantages, so the characteristics of the raw material and the actual production requirements should be taken into account when selecting a method for preparing the protein.

**Table 2:** Walnut protein preparation methods and their advantages and disadvantages.

Preparation methods	Principle	Advantages	Disadvantages	Reference
alkali-solution and acid isolation	Separation using the difference in acidity and alkalinity of the components in the raw material mixture	The purity of the isolated protein is up to 90% or more, the quality of the product is good and it is the most widely used in practice.	The removal of soluble components is not complete and the acid solution consumed is high.	(24)
Salt dissolution and acid deposition method	Adjust the pH of the raw material mixture to precipitate the walnut protein near the isoelectric point.	High extraction rate, simple operation, and low cost.	The protein obtained has more impurities.	(24)
Ethanol leaching method	A certain concentration of ethanol is used to wash the walnut residues to denature the walnut protein, which loses its solubility and precipitates the walnut protein.	It has some feasibility.	Reduces the solubility of the protein by denaturation, which is not conducive to subsequent processing.	(25)
reversed micelle method	The solubilization properties of the inverse micelles (oil-in-water microemulsions) are used to dissolve the walnut proteins in the polar nuclei.	The spatial structure of plant proteins is preserved to a greater extent.	In the extraction of large molecular mass proteins, different degrees of physical changes occur and the extraction conditions have a strong influence on the shape of the protein	(26)
Membrane separation	Separation of low molecular proteins using RO (Reverse Osmosis) membranes	Low energy consumption, simple operation, no pollution	However, ultrafiltration membranes are susceptible to contamination, which can affect changes in protein properties	(24)
Ion exchange method	Modulation of solution pH values by ion exchange, resulting in protein leaching and precipitation	High protein purity	Long production cycles	(27)
Subcritical extraction	Separation of proteins by the principle of similar solubility using subcritical water as an extractant	Improved extraction efficiency and greatly reduced extraction times	Protein degradation due to high temperature	(27)

#### 3.1.2 Bioactivity of walnut vegetable proteins

Walnut protein has biological activities such as antioxidant and anti-inflammatory. Han Haitao et al. showed that the main components of Walnut protein had good antioxidant activity, and the DPPH radical scavenging ability of the clear protein, alcoholic protein, and gluten-2 in walnut protein could reach 97.15%, 93.35%, and 90.58%, respectively (24). Meanwhile, Walnut protein delayed the onset of acute colitis induced by sodium dextran sulfate, slowed down the weight loss in mice caused by colitis, and had significant anti-inflammatory activity due to the hydrolysis of walnut protein into small molecule peptides with anti-colitis activity by the action of intestinal digestive enzymes, which exerted anti-inflammatory effects, which inspired us to focus our research on the biological activity of walnut peptides (12).

### 3.2 Walnut peptides

#### 3.2.1 Functional properties of walnut peptides

**Figure 5:**
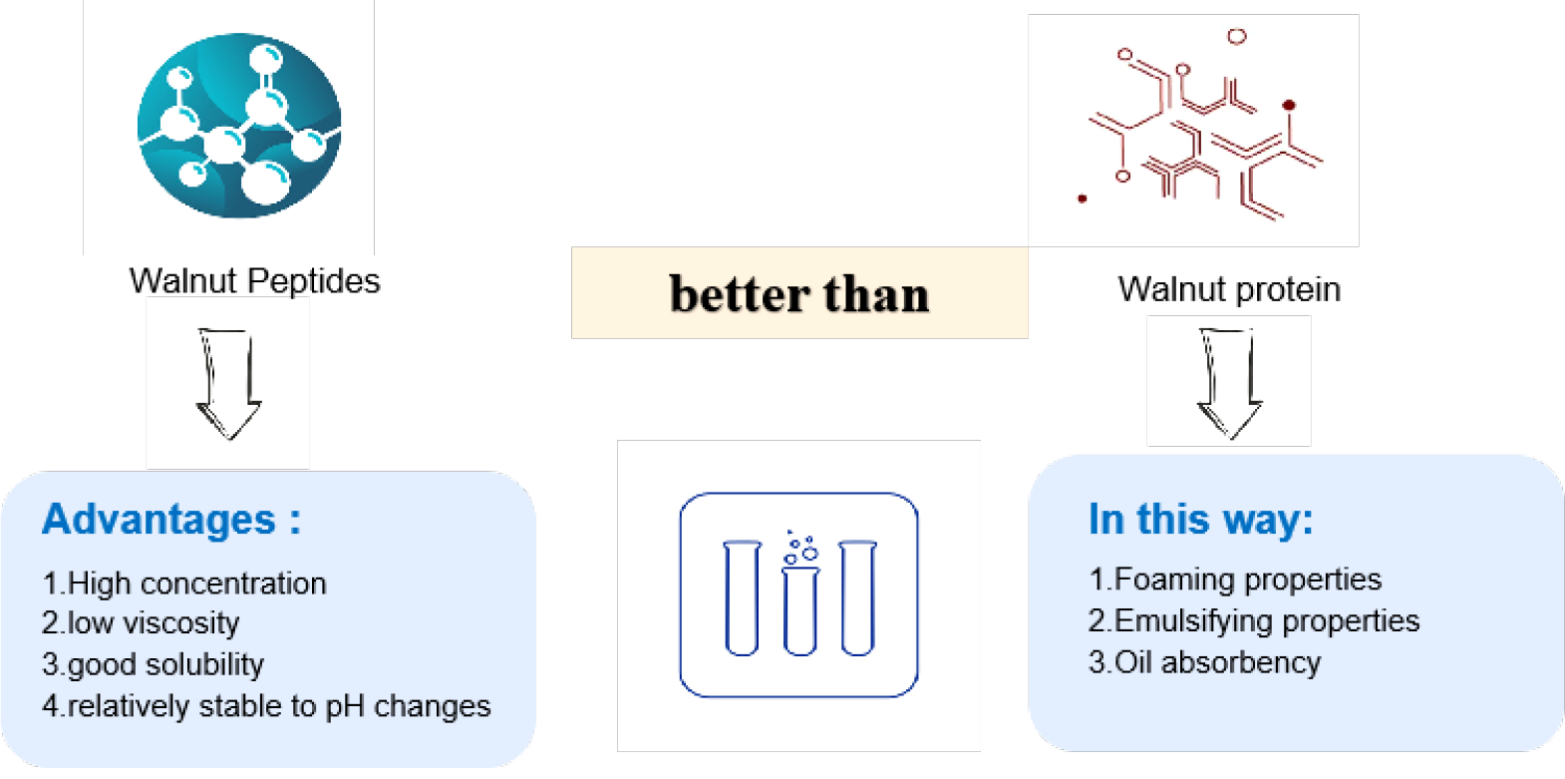
Benefits of Walnut Peptides.

Numerous studies have confirmed that walnut peptides have a strong antioxidant capacity both *in vitro* and *in vivo*, which is closely related to the amino acid sequence and composition of the peptides (31, 32). Walnut peptides are mostly composed of two to several dozen amino acids through peptide bonds and their relative molecular mass is generally less than 6000 Da (30). As a natural active peptide, the walnut peptide has good characteristics such as high concentration, low viscosity, good solubility, and relative stability to pH changes, and it is better than Walnut protein in terms of foaming, emulsification, and oil absorption, with high safety and excellent application prospects (**Figure 5**).

#### 3.2.2 Walnut peptides are mainly prepared by enzymatic digestion

Walnut peptides have a variety of biological activities, however, whether the peptides can perform normally or maximize their biological activity requires the selection of a suitable preparation and extraction method according to the characteristics of the raw material, the actual production needs, and the available conditions. The preparation of walnut peptides refers to the process of hydrolysis of walnut vegetable proteins into small molecular peptides with molecular weights between amino acids and proteins using biological or chemical methods. Bioactive peptides are prepared from plants by enzymatic, fermentation, and chemical methods (33). Enzymatic and fermentation methods are more commonly used (34, 35) (**Table 3**), while enzymatic digestion is the most dominant method of preparation (39).

**Table 3:** Comparison of walnut peptide preparation methods.

Preparation methods	Enzymatic digestion	Fermentation method	Chemical method	Reference
Definition	Proteolytic preparation of bioactive peptides is a process that uses proteases to hydrolyze proteins to obtain an enzymatic solution, which is then separated and purified to obtain bioactive peptides.	Microbial fermentation for the preparation of active peptides is a method for the orderly degradation of macromolecular proteins through the extracellular enzyme system produced by the beneficial strains themselves to produce various peptides.	Chemical hydrolysis is a method of producing active peptides by breaking the peptide bonds of proteins by bathing them in an acid or base solution at the appropriate concentration for some time and at the appropriate temperature.	(36)
Advantages	Mild reaction conditions, safe operation, mature research, directional enzyme catalytic position, and no stereoisomerization or racemization, suitable for mass production	Simple handling, no solvent residues, low processing costs, and a de-bittering effect.	Easy to operate and inexpensive.	(37, 38)
Disadvantages	However, there are ionic introductions in the preparation process and the peptides are inevitably colored by the Merad reaction during fermentation, and the hydrophobic peptides produced during hydrolysis can also give the peptides an undesirable flavor (bitterness, and odor), limiting their practical application.	The method requires strain screening, cultivation, longer fermentation cycles, more complex enzyme systems produced by metabolism, and more hydrolysis by-products, and is limited by the safety of the strain used, so microbial fermentation methods are difficult to expand.	However, the extraction rate is low, the reaction process is not easily controlled, the product quality is unstable, the environmental pollution is more serious and the economic efficiency is low, which is not suitable for modern industrial production of walnut peptides.	(34)

##### 3.2.2.1 Enzymatic preparation of walnut peptides

Walnut residue has 15% to 20% residual oil, which leads to protein denaturation unfavorable to storage, and how to degrease it is an urgent technical problem to be solved (40). Leaching is the main method used to degrease walnut residues; however, this method has solvent residues and is complex to operate. In recent years, subcritical extraction techniques have been increasingly used in the extraction of oil and fats, and are available on a large scale for industrial production (41).

In the preparation of walnut peptides, the choice of enzymes is crucial. The commonly used enzymes and their enzymatic effects are alkaline proteases> papain > trypsin > neutral protease > flavor protease > pepsin (34, 42, 43). Wang Duan (44) et al. used neutral protease to hydrolyze defatted walnut residue powder to prepare peptides and optimized the extraction process of walnut peptides, and the peptide yield was 0.45 g/g under the optimal process conditions. Lu Xiaodan (45) showed that microwave and ultrasonic treatment resulted in significantly higher yields of walnut peptides. Chen Shujun (46) et al. used a complex protease enzyme to optimize the complex protease enzymatic digestion process, and the peptide mass concentration was 10.01 mg/mL and the degree of hydrolysis was 11.45% under the optimal enzymatic conditions.

The above studies illustrate that when preparing walnut peptides by enzymatic digestion, the preparation efficiency and biological activity are influenced by the conditions of enzymatic digestion, the type of complex enzyme, and the sequence of enzymatic digestion and pretreatment. Enzymatic digestion is still the most important method for the preparation of walnut peptides now. To obtain good biologically active walnut peptides efficiently, suitable proteases should be selected according to the actual production needs, providing a favorable guide for the industrial production of walnut peptides.

The exposure of hydrophobic amino acids of the protein after enzymatic digestion causes bitterness limiting its application in food (47). Choosing the right method of de-bittering can improve the taste and flavor of the product and increase its usefulness in processing and production. Three methods are currently commonly used to remove the bitterness of proteinaceous peptides (41) (**Table 4**).

**Table 4:** Comparison of the advantages and disadvantages of the three methods of debittering.

Methods	Advantages	Disadvantages	Reference
Selective adsorption	Environmentally friendly, safe, and cost-effective	Loss of some amino acids, reducing nutritional value	(41)
Enzymatic method	No loss of nutrients	Further alteration of the molecular structure of the short peptide chain affects its functional properties and is costly and unsuitable for industrial production	(41)
Cover-up method	No nutrient loss, low cost	A sufficient amount of masking agent needs to be added, which may cause odors.	(41)

##### 3.2.2.2 Preparation of walnut peptides by fermentation

Fermentation methods are divided into solid and liquid fermentation, with solid fermentation being a microbial fermentation process that takes place on a solid substrate feedstock with little to no free-flowing water in the fermentation substrate (48). Compared to liquid fermentation, solid-state fermentation is less costly, has a wider source of substrates, is less polluting to the environment, is less technically and environmentally demanding, and is more operational (49). Liquid fermentation, with its high level of free water, is a relatively new fermentation technology that began in 1995 (50). Liquid fermentation makes full use of raw materials, and has a short fermentation cycle and a stable product quality, but requires advanced and well-controlled production equipment and a large investment in equipment.

Walnut peptides were mostly prepared by solid-state fermentation of walnut residues. He Ying (51) et al. used lactic acid fermentation of walnut meal to prepare walnut peptides, and the yield of walnut peptides reached 12.84 mg/g and had a powerful free radical scavenging rate and reducing ability. Liu Xiao (52) et al. used Bacillus subtilis and Aspergillus niger for solid-state fermentation of walnut residues, and the result was that Bacillus subtilis fermentation produced a significantly higher content of walnut peptides than Aspergillus niger at 243.97 mg/g. Wu Wanxing (53) used fungal and bacterial microorganisms for solid-state fermentation of walnut residues and concluded that the bacterial peptide yield was significantly higher than the fungal at 30.20% at the respective optimum fermentation temperatures.

Liquid fermentation has the advantage of a shorter fermentation cycle than solid fermentation, faster cell appreciation, more uniform cell development, and so on. Liang Heng (54) et al. screened Bacillus subtilis, Bacillus natto, and Bacillus licheniformis from Bacillus subtilis for liquid fermentation of walnut meal, The fermentation process was also optimized to verify the inhibitory effect of the polyphenolic substances in walnut residues on the growth of Bacillus subtilis and showed a concentration dependence. Using liquid fermentation technology, Xu Dian (55) investigated the optimal process parameters for the fermentation of Aspergillus niger and determined the antioxidant capacity of four constituents of walnut peptides with different molecular weights. The experimental results showed that the highest peptide content of 7.20 mg/mL was achieved at the optimum process parameters, and the antioxidant capacity of the fermentation liquid was significant, with walnut peptides with a molecular weight of <5 KDa having the strongest antioxidant capacity.

##### 3.2.2.3 Isolation and purification of walnut peptide molecules

Studies have shown that the antioxidant activity of peptides is closely related to their molecular weight (56, 57), and the smaller the molecular weight, the stronger the antioxidant activity of short peptides (32, 46). Therefore, increasing the degree of hydrolysis when carrying out hydrolysis, adding small molecular weight peptides, and separating and purifying the enzymatic digest maximize the biological activity of walnut peptides.Common active peptide isolation methods (**Table 5**) (33, 58).

**Table 5:** Classification and characteristics of peptide isolation and purification methods.

Isolation and purification methods		Advantages	Disadvantages	Effect	Applied	Reference
Membrane Separation Technology	Ultrafiltration	Easy to operate, low cost, energy saving, and environmental protection, conducted at normal temperature, avoiding the destruction of active peptides by high temperature, effectively retaining the activity of active peptides	Not suitable for the isolation of a specific peptide	Better	Suitable for primary separation, separation of medium to large mixtures	(33)
	Nanofiltration	Separation process without adding any chemical reagents, no heating, no phase change, good desalination effect	Research on the design of nanofiltration membrane materials, membrane performance, and structure is not mature enough	Good	Widely used in industrial production	(33)
Chromatography	Reversed-phase high-performance liquid chromatography	High resolution, high sensitivity, good separation, simple operation, automatic analysis of target peptides in large batches	Expensive instrumentation and insufficient retention of hydrophilic small molecule peptides	Good	Wide applicability, especially for peptides with small molecular masses	(33)
	Gel filtration chromatography	Easy handling, mild operating conditions, no organic solvents required, no effect on the activity of the active peptides	The separation operation is generally slow and difficult to achieve good separation of active peptides with similar molecular weights	Good	Wide applicability for the separation of active peptides	(33)
	Ion-exchange chromatography	High sensitivity, good selectivity, and fast analysis	The ionic strength of the eluent, salt concentration, and other influences, and the elution may introduce impurity ions and need to be desalted again	Good	Wider applicability	(33)
	Affinity chromatography	High selectivity and good separation	More expensive carriers, adsorption of neuropeptides	Good	The small range of applications and high specificity for the isolation and purification of glycopeptides	(33)
salting-out method		Easy and convenient, low cost	The pure concentration is not high and introduces a lot of salt, which needs to be desalted	Better	Suitable for initial purification only	(58)
Electrophoresis		Low sample volume, high sensitivity, fast analysis, and high separation efficiency	Low feed volume makes mass production difficult	Good	Widely used in active peptide isolation	(58)
the aqueous two-phase extraction technique		The isolated peptides have fewer impurities, do not change the activity and conformation of the peptides, and are less toxic and biocompatible	Water-soluble polymers are difficult to recover, non-volatile, and easily emulsified	Good	Wide applicability for the separation of active peptides	(58)

Several methods are available for the isolation of bioactive peptides, each with its advantages and disadvantages, and often a single isolation method does not meet the requirements. Thus, to obtain highly active, high-purity target peptides, multiple isolation methods are often used in combination.

##### 3.2.2.4 Others

Walnut seed coat is rich in polyphenols, which can be reacted with browning under the action of polyphenol oxidase, forming brown compounds that seriously affect the quality of walnut further processed products. Cheng Jing (59) used 2% citric acid for pretreatment in the preparation of walnut peptides from walnut residues, which reduced the browning reaction. Decolourisation also plays a crucial role in the preparation of walnut peptides. Wang Wei (60) et al. explored the optimal conditions for the decolorization of enzymatic hydrolysate of walnut protein by activated carbon, and the decolorization rate was 78.05% under these conditions. According to the actual needs of the selection of suitable methods of polyphenol removal and decolorization, the process can improve the quality and market competitiveness of the product and broaden the development of walnut peptides.

#### 3.2.3 Walnut peptide bioactivity

The conventional concept is that proteins are ingested and absorbed by the body after being hydrolyzed into amino acids by various proteases, however, the discovery of peptides and peptide absorption channels in the small intestine suggest that proteins are not necessarily absorbed as free amino acids after digestion and degradation, rather they are mainly in the form of oligopeptides (61), that is, small molecules of peptides are more easily absorbed than proteins or amino acids (42). The biological activities and mechanisms of action of walnut peptides in gut microbiota regulation, antioxidants (8, 34, 42), anti-fatigue, memory improvement, anti-epilepsy, and improvement of metabolic diseases have been investigated by domestic and foreign researchers.

##### 3.2.3.1 Gut microbiota regulation

It’s shown that the walnut-derived peptide leucine-proline-phenylalanine (LPF) has a protective and restorative effect on dextran sodium sulfate-induced colitis in mice. Besides, the walnut peptide regulated the gut microbiota disorders in mice by increasing the relative abundance of beneficial genera and decreasing the relative abundance of potentially harmful genera (62). Wang et al. showed the effect of walnut-derived peptide PW 5 on Beta-amyloid protein and intestinal microbiota (63). The above studies have verified the role of walnut peptides in gut microbiota regulation.

##### 3.2.3.2 Antioxidant activity

Qian Du (64) et al. proposed the hypothesis that walnut peptides may have functions such as promoting brain development and improving learning and memory by significantly increasing the antioxidant properties of tissues and enhancing the ability for free radical scavenging. The mechanisms of protective effects of three novel nucleotides, LVRL, LRYL, and VLLALVLLR, on high glucose-induced insulin resistance (IR) and oxidative stress in HepG 2 cells were elucidated by Wang et al (65). Walnut-derived peptides significantly reduce reactive oxygen species (ROS). Walnut-derived peptides LVRL and LRYL increase antioxidant enzyme activity pathways by activating Nrf2/HO-1 signaling, thereby inhibiting high glucose-induced ROS production and MAPK activation and improving glucose uptake and IR in HepG 2 cells, resulting in alleviating effects on hepatic IR, probably due to the reduced oxidative stress characteristics of walnut residues.

##### 3.2.3.3 Memory improvement

Alzheimer’s disease(AD) is one of the major neurodegenerative diseases in the elderly, with Aβ-induced oxidative stress and neuroinflammation in the brain attributing to the pathogenesis of AD. Zhao et al. showed (66) that the walnut peptide YVLLPSPK improved learning and memory in scopolamine-induced cognitive impairment in mice through a mechanism related to the NF2/KEAP1/HO-1 pathway, providing a deeper theoretical basis for the research on the mechanisms by which walnut peptides improve learning memory and cognitive impairment. Zou et al. (67) demonstrated that the addition of walnut peptides was effective in improving cognitive impairment and memory disorder in mice and that supplementation with walnut peptides was effective in restoring the levels of antioxidant enzymes and inflammatory mediators, thereby reducing the inflammatory response and regulating the antioxidant systems et al. (67) demonstrated that the addition of walnut peptides was effective in improving cognitive impairment and memory deficits in mice, and that supplementation with walnut peptides was effective in restoring levels of antioxidant enzymes and inflammatory mediators, thereby reducing inflammatory responses and modulating the antioxidant system which has a protective effect on AD.

##### 3.2.3.4 Anti-fatigue

It’s proved that walnut peptide alleviates fatigue by promoting the synthesis of red blood cells in the animal body so that it could reduce the production of lactic acid and urea ammonia during strenuous exercise and delay fatigue. At the same time, it can rapidly decompose lactic acid and urea ammonia and expel them out of the body after exercise, thus speeding up fatigue recovery (68, 69). Liu et al. showed (8) that walnut oligopeptide significantly inhibited fatigue-induced oxidative stress, improved pyruvate kinase, and succinate dehydrogenation activities in mouse skeletal muscle increased mitochondrial biogenesis factor mRNA expression and mitochondrial content and exerted anti-fatigue effects in mice. Uran et al. (47) measured the time to exhaustion of weighted swimming, serum urea nitrogen and lactate dehydrogenase activity in each group of mice, the blood lactate level in each group of mice, and the liver and muscle glycogen content in each group of mice. The results showed that walnut peptides increased lactate dehydrogenase activity, reduced blood lactate and serum urea nitrogen levels, increased muscle glycogen reserves, and significantly prolonged weight-bearing swimming time, thus having a better anti-fatigue effect. It’s shown that, as a plant-derived active peptide, walnut relieve fatigue to some extent.

##### 3.2.3.5 Other activities

Walnut peptides still have other functions, such as antiepileptic activity, improving hyperlipidemia as well as hepatic lipid metabolism, and so on (**Table 6**).

**Table 6:** Other activities of walnut peptides.

Active	Research Progress	Reference
Antiepileptic activity	Jahanbani et al. evaluated the antiepileptic properties of walnut peptide extracts in three different mouse seizure models (pentylenetetrazol-induced clonic seizures, chemical ignition, and maximal electroshock). The experimental results showed that intraperitoneal administration of walnut peptides significantly increased the seizure threshold; walnut peptides exert their antiepileptic properties through the modulation of benzodiazepine receptors.	(62, 70)
Improves hyperlipidemia and hepatic lipid metabolism	Rats fed walnut peptide powder counteracted the high-fat-induced increase in body, liver, and epididymal fat weight and could lower total serum cholesterol, triglycerides, and total low-density cholesterol, raise high-density lipoprotein cholesterol and lower the atherosclerotic index. In terms of body weight, the total energy intake of rats in the walnut peptide group was close to that of rats on a high-fat diet, but the body weight of rats in the walnut peptide-treated group was significantly lower than that of rats on a high-fat diet, so that intake of walnut peptides was effective in improving hyperlipidemia and liver lipid metabolism disorders.	(63, 71–73)
Anti-cancer activity	Ma et al. used papain to hydrolyze walnut meal to obtain a novel biopeptide with the amino acid sequence CTLEW and investigated the anti-cancer mechanism, showing that the CTLEW peptide could induce apoptosis and autophagy in MCF-7 cells and exhibited selective inhibition and immunomodulatory activity against cancer cell growth.	(64, 74)
Laxative for bowel movement	Zhang Ting et al. explored and verified the effect of walnut peptides in improving constipation and laxative effect, and explored the mechanism by which the effect occurred. The researchers inferred that the mechanism of the action of walnut peptides in conjunction with the experimental results may be related to the fact that walnut oligopeptides promote the expression of gastrointestinal hormone endotoxin and gastrin in the serum of mice and inhibit the expression of growth inhibitors.	(65, 75)

## 4 Conclusion

The two applications of walnut plant-based are considered in terms of economic benefits, nutritional value, and environmental impact, each with its focus. Walnut plant protein powder is cheaper to produce but has less nutritional and food applications than walnut peptides, while walnut peptides, although slightly more costly than walnut plant protein, have a variety of biological activities and are widely used in food and pharmaceuticals, and researchers have focused on walnut peptides. In recent years, the rich walnut resources of Yunnan have received a great deal of attention from researchers. The application of plant-based products in food has also created a boom in international and domestic markets. Plant-based dairy products, plant-based meat, and other plant-based products are entering the market. Research on walnut plant protein powder and walnut peptides has also focused on their active functions such as antioxidant, anti-fatigue, and memory improvement, and the subsequent development of healthy food and functional food with various functions by combining walnut plant protein powder and walnut peptides with herbs in Yunnan. For example, walnut peptides can be compounded with herbs in Yunnan such as *Gastrodia elata Bl*., *Polygonati Rhizoma*, and *Radix Puerariae* (76–78) (**Table 7**) to develop healthy foods and functional foods with anti-fatigue, immunity enhancement, and unique flavors. However, due to Yunnan’s backward economic and technological level, the walnut resources are mostly in primary processing products, the walnut plant-based resources are discarded and wasted, and the visibility is low. Consequently, it is not only necessary to combine walnut plant-based products with Yunnan’s authentic medicinal herbs to improve their innovation, but also to actively improve the production methods to open up the visibility of Yunnan’s walnut plant-based products by joining forces with the government, the public and the media, so that Yunnan’s walnut resources and walnut plant-based products go out of Yunnan and out of China and that the walnut plant-based products of Yunnan have a place in the plant-based market at home and abroad.

**Table 7:** Chinese and Latin names of Chinese medicines.

Chinese name	Chinese Pinyin name	Latin name
天麻	Tianma	*Gastrodia elata Bl*
黄精	Huangjing	*Polygonati Rhizoma*
葛根	Gegen	*Radix Puerariae*

## Author contributions

Author contributions were as follows: study design YW and HX, data collection XZ, XP and HP, data interpretation YC, HM and JY, manuscript preparation XZ, XP and HP, and funds collection JY. All authors contributed to the article and approved the submitted version.
